# Standardization and Evaluation of an Anti-ZIKV IgM ELISA Assay for the Serological Diagnosis of Zika Virus Infection

**DOI:** 10.4269/ajtmh.21-0163

**Published:** 2021-08-02

**Authors:** Kanittha Sirikajornpan, Piyarat Suntarattiwong, Detchvijitr Suwanpakdee, Sutchana Tabprasit, Darunee Buddhari, Butsaya Thaisomboonsuk, Chonticha Klungthong, Yongyuth Poolpanichupatam, Rome Buathong, Anon Srikiatkhachorn, Anthony Jones, Stefan Fernandez, Taweewun Hunsawong

**Affiliations:** ^1^ Department of Virology, Armed Forces Research Institute of Medical Sciences (AFRIMS), Bangkok, Thailand;; ^2^ Pediatrician, Infectious Diseases Unit, Department of Pediatrics, Queen Sirikit National Institute of Child Health, Bangkok, Thailand;; ^3^ Department of Pediatrics, Phramongkutklao Hospital, Bangkok, Thailand;; ^4^ Research Division, Royal Thai Army-Armed Forces Research Institute of Medical Sciences (RTA-AFRIMS), Bangkok, Thailand;; ^5^ Department of Disease Control, Bureau of Epidemiology, Ministry of Public Health, Nonthaburi, Thailand;; ^6^ Institute for Immunology and Informatics, University of Rhode Island, Providence, Rhode Island;; ^7^ Faculty of Medicine, King Mongkut’s Institute of Technology Ladkrabang, Bangkok, Thailand

## Abstract

Here, we describe the development of the in-house anti-Zika virus (ZIKV) IgM antibody capture ELISA (in-house ZIKV IgM ELISA) for the detection and diagnosis of acute ZIKV infections. We compared the in-house ZIKV IgM ELISA assay performance against two commercial kits, Euroimmun ZIKV IgM and InBios 2.0 ZIKV IgM ELISA. We tested the assays’ ability to detect anti-ZIKV IgM using a well-defined serum sample panel. This panel included 80 ZIKV negative samples (20 negative, 20 found to be primary dengue virus [DENV][ infections, 20 secondary DENV infections, and 20 Japanese encephalitis virus [JEV] infections) and 67 ZIKV reverse transcriptase–polymerase chain reaction–positive acute serum samples. The OD values were calculated to enzyme immunoassay (EIA) unts by comparing them to weak positive controls. The results demonstrated the high sensitivity (88.06%) and specificity (90.00%) of our in-house ZIKV IgM ELISA and its 89.12% overall percentage agreement. The kappa values were deemed to be within excellent range and comparable to the InBios ZIKV IgM ELISA. Some cross-reactivity was observed among secondary DENV and JEV samples, and to a much lower extent, among primary DENV samples. These data indicate that our in-house ZIKV IgM ELISA is a reliable assay for the detection of anti-ZIKV IgM antibodies in serum.

## INTRODUCTION

Zika virus (ZIKV) is a mosquito- borne pathogen, belonging to the *Flaviviridae* family, in the Spondweni sero-complex group.^1^ Zika virus was first isolated from a sentinel Rhesus monkey (*Macaca mulatta*) during a yellow fever study in the Zika forest near Entebbe, Uganda, in 1947.^2^ In 1948, the virus was isolated from pooled *Aedes africans* circulating in the same forest.^3^ Zika virus has also been isolated from many other species of *Aedes* mosquitoes,^4^^,^^5^ including *Aedes aegypti* mosquitoes,^6^ the most significant vector of ZIKV transmission. Human-to-human transmission can also occur through blood transfusions, sexual contact, and vertically from mother to fetus.^7^

The first ZIKV infection in humans was reported in Nigeria in 1954.^8^ Zika virus infections usually manifest as asymptomatic or mild disease, most commonly accompanied by mild fever, arthralgia in small joints of the hands and feet, myalgia, headache, retro-orbital pain, conjunctivitis, and cutaneous maculopapular rash. Clinical diagnosis is often difficult because symptoms are shared by infections with other arboviruses, like dengue virus (DENV) and chikungunya virus (CHIKV). Abdominal pain, diarrhea, and arthritis can also appear in some cases of ZIKV infections.^9^ In 2007, a large outbreak of ZIKV disease took place in Yap state, Micronesia, infecting approximately 70% of the population.^10^ Since then, ZIKV has spread throughout various regions of the world,^11^^–^^13^ becoming a significant public health threat due to its association with significant neurological disorders in infants.^14^^–^^16^ Given the persistent circulation of ZIKV in some areas of the world, including Thailand,^17^ improvements in early detection and responses, vector control programs, effective therapeutics and vaccines are needed to control infection and transmission.^18^

The laboratory diagnosis of ZIKV mostly relies on the detection of viral RNA in whole blood (also serum and plasma), cerebrospinal fluid, saliva, urine, and semen.^19^^,^^20^ Zika virus viremia can be detected for up to 5 days after symptoms onset, peaking when clinical signs appear.^10^ Some evidence shows longer detectable periods of ZIKV viremia in urine and semen than in whole blood or saliva.^21^^,^^22^ Serological tests like ELISAs can provide a wider window for diagnosis because they are capable of detecting ZIKV antibodies’ response early during an acute event and through convalescence. Zika virus IgM typically develops around 5 days after symptom onset and remains detectable for at least 12 weeks, whereas ZIKV IgG can be detected a few days later and remains detectable for at least 1 year.^20^ Despite its high cross-reactivity to other flaviviruses,^23^ detection of circulating ZIKV IgG antibodies continues to be widely used to identify prior ZIKV exposures in individuals.^24^ We seek to test the less cross-reactive ZIKV IgM antibodies as a tool to distinguish acute ZIKV infections from other flavivirus and extend the diagnostic window provided by reverse transcriptase–polymerase chain reaction (RT-PCR). Here, we standardized and characterized the performance of the in-house ZIKV IgM ELISA using patient sera and compared it with two commercial ELISA kits including Euroimmun ZIKV IgM ELISA (Euroimmun ELISA; Euroimmun AG, Lübeck, Germany) and InBios ZIKV detect 2.0 IgM capture ELISA (InBios MAC-ELISA; InBios international, Inc., Seattle, WA).

## MATERIALS AND METHODS

### Serum specimens.

The human serum samples used in this study (Table 1) were obtained from Thai patients and confirmed by RT-PCR under a non-human subject research study approved by Walter Reed Army Institute of Research and local Institutional Review Boards. The deidentified samples consisted of a total of 147 pairs of acute and convalescent samples, including 67 ZIKV RT-PCR–positive samples and 80 ZIKV RT-PCR–negative samples. The RT-PCR–negative samples were composed of samples with no evidence of flavivirus infection (*N* = 20), primary DENV infections (*N* = 20), or secondary DENV infections (*N* = 20) and were Japanese encephalitis virus (JEV) IgM positive (*N* = 20).

**Table 1:** Sample panel characteristics of *N* = 147 cases in pairs used in the study

Group	Label	Cases (*N* = 147)	RT-PCR			DENV/JEV MAC-ELISA	
			DENV	JEV	ZIKV	DENV	JEV
1	Negative	20	0	0	0	0	0
2	1° DENV	20	20	0	0	20	4
3	2° DENV	20	20	0	0	20	0
4	JEV	20	0	0	0	0	20
5	ZIKV	67	0	ND	67	16	8

DENV = dengue virus; JEV = Japanese encephalitis virus; MAC-ELISA = IgM antibody capture enzyme-linked immunosorbent assay; ND = not determined; RT-PCR = reverse transcriptase–polymerase chain reaction; ZIKV = Zika virus.

### Viral RNA detection by RT-PCR.

Viral RNA was extracted using QIAamp viral RNA mini kit (Qiagen, Hilden, Germany) according to the manufacturer’s instruction. Samples were tested by nested RT-PCR for detection of DENV and JEV^25^ and real-time RT-PCR for detection of ZIKV. For ZIKV real-time RT-PCR, the method was modified from Lanciotti et al.^20^ by using the two primer/probe sets. The first set includes ZIKV 1086 forward primers, ZIKV 1162c reverse primers, and a ZIKV 1107-FAM probe,^26^ and the second set includes ZIKV 4434 forward primers, ZIKV 4524c reverse primers, and a ZIKV 4479c-FAM probe.^27^ All real-time assays were performed by using the SuperScript III Platinum One-Step Quantitative RT-PCR System (Invitrogen, Waltham, MA) with amplification using the Applied Biosystems 7500 Fast Real-Time PCR systems (Life Technologies, Carlsbad, CA) following the manufacturer’s protocol.

### Anti-DENV/JEV IgM and IgG ELISA.

Anti-DENV/JEV IgM and IgG ELISAs were performed in three independent experiments following procedure described elsewhere.^28^^,^^29^ Briefly, flat-bottom microplates were coated with 100 μL/well of 1:1,600 dilution of goat anti-human IgM or IgG (KPL, Gaithersburg, MD) in 0.018 M carbonate buffer (pH 9.0). After overnight incubation at 4°C, the plates were washed with phosphate buffered saline (PBS) (pH 7.4) containing 0.5% Tween 20 (PBS-T). Next, 50 μL/well of 1:100 dilution of test serum, negative control (NC), weak positive control (WPC), and strong positive control (SPC) in PBS were added and incubated overnight at 4°C. After washing with PBS-T, 50 μL/well of sucrose acetone extracted suckling mouse brain DENV (pooled DENV antigen: DENV-1 [Hawaii], DENV [NGC], DENV-3 [H87], and DENV-4 [H241]) and JEV (JaGAr01) antigens were added into DENV (IgM/IgG) and JEV (IgM/IgG) plates, respectively. After incubation for 2 h at room temperature, 30 μL/well of human anti-flavivirus IgG–horseradish peroxidase conjugated was added and incubated for 1 hour at 37°C. After washing with PBS-T, 100 μL/well of TMB substrate (KPL, Gaithersburg, MD) was added and incubated for 10–30 min. The reaction was stopped by adding 50 μL/well of 0.2 M sulfuric acid. The absorbance (optical density [OD]) was measured at a wavelength of 450 nm (SoftMax Pro Software, Molecular Devices, San Jose, CA). A valid assay should provide OD values at < 0.100, 0.400–0.600, and > 0.600 for NC, WPC, and SPC, respectively. EIA units of tested serum are equal to 100 × [(OD_Test_ − OD_NC_)/(OD_WPC_ − OD_NC_)]; EIA units of IgM ≥ 40 were used as a positive cut-off value. Evidence of dengue infection was classified by a ratio of DENV IgM/JEV IgM ≥ 1.0, and JEV infection when the ratio was < 1.0. Primary DENV infection was interpreted when the ratio of DENV IgM/DENV IgG was ≥ 1.8, and secondary DENV infection was considered when the ratio was < 1.8.

### In-house ZIKV IgM ELISA.

A capture ELISA method was used to develop an in-house ZIKV IgM ELISA. Briefly, flat-bottom microplates were coated with 100 μL/well of 1:1,600 dilution of goat anti-human IgM (KPL, Gaithersburg, MD) in 0.018 M carbonate buffer (pH 9.0). After overnight incubation at 4°C, the plates were washed with PBS (pH 7.4) containing 0.5% Tween 20 (PBS-T). Next, 50 μL/well of 1:100 dilution of test serum, NC, WPC and SPC in PBS were added and incubated overnight at 4°C. After washing with PBS-T, 50 μL/well of sucrose acetone extracted suckling mouse brain ZIKV (MR766; Uganda, 1947) antigen (50–100 HA units) was added to the microplate. After incubation for 2 h at 37°C, 30 μL/well of human anti-flavivirus IgG–HRP conjugated was added and incubated for 1 h at 37°C. After washing with PBS-T, 100 μL/well of TMB substrate (KPL) was added and incubated for 10–30 min. The reaction was stopped by adding 50 μL/well of 0.2 M sulfuric acid. The absorbance at OD450 was measured and calculated for EIA units as previously described.^28^^,^^29^ The WPC for anti-ZIKA IgM ELISA was obtained from the serum of a rhesus monkey (*Macaca mulatta*) 15 days after inoculation with 5 × 10^6^ PFU of ZIKV (MR766; Uganda, 1947).

### Euroimmun anti-Zika virus IgM ELISA (Euroimmun ZIKV-IgM).

The Euroimmun ZIKA IgM ELISA test kit was designed to detect specific IgM against recombinant ZIKV NS1 coated on the plate. The Euroimmun Zika Virus ELISA kits (Cat. No. EI2668-9601M) were produced by Euroimmun AG (Lübeck, Germany). The assays were carried out according to the manufacturer’s instructions.^30^

### InBios ZIKV detect^TM^ 2.0 IgM capture ELISA (InBios ZIKV-IgM).

The InBios ZIKV IgM ELISA detects IgM antibodies targeting the recombinant ZIKV envelope glycoproteins (Cat. No. ZKM2-1). The InBios ZIKV-IgM ELISA kit was produced by the InBios international, Inc. (Seattle, WA) and performed according to the manufacturer’s instructions.^31^

### Data analysis.

Prism-GraphPad (GraphPad software Inc., La Jolla, CA) was used to create the receiver operating characteristic (ROC) curve to identify an appropriate cut-off value for anti-ZIKV IgM ELISA. The assay performance comparison to commercial kits (assay agreement) was evaluated by Kappa values, indicating excellent agreement if > 0.75, fair agreement if 0.40–0.75, and poor agreement if < 0.40.^32^

## RESULTS

### Receiver operating characteristic curve of anti-ZIKV IgM ELISA.

**Figure 1.**
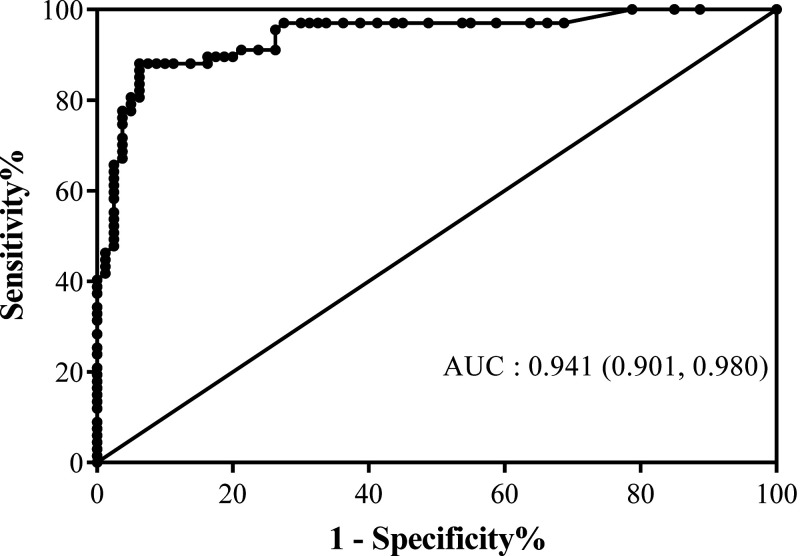
Receiver operating characteristic (ROC) curve of the In-house ZIKV IgM ELISA. The graph shows the area under the ROC (AUC) value and the 95% confidence intervals in parentheses. The sensitivity and specificity values correspond to the points in the plots. The ROC curve was constructed using 80 ZIKV RT-PCR negative and 67 cases of ZIKV RT-PCR positive samples.

**Figure 2.**
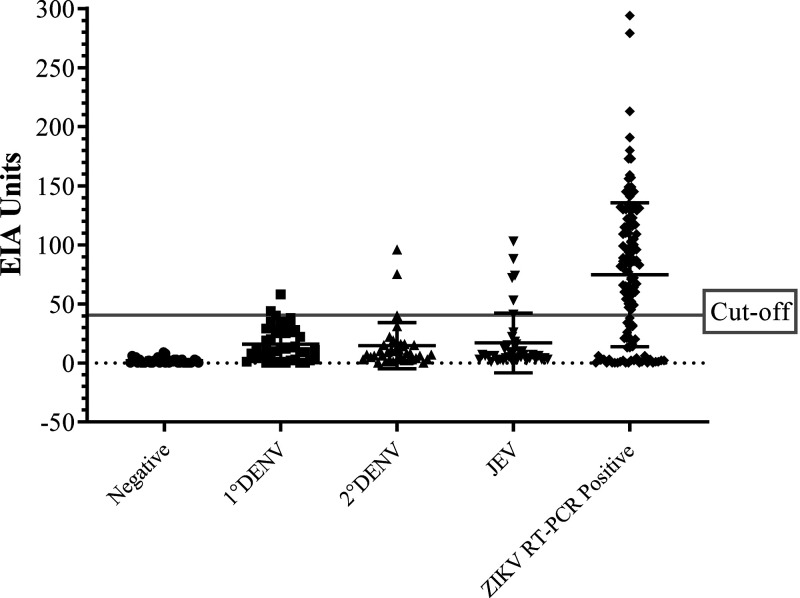
The EIA unit distribution of validated samples by using the In-house ZIKV IgM ELISA. The cut-off was set at 40 EIA unit.

Table 1 shows the composition and characteristics of the serum panel used. All of the samples were tested by RT-PCR and DENV/JEV IgM/IgG ELISA. The ROC curve (Figure 1) was used to determine an optimal EIA unit cut-off point to distinguish between negative and positive samples and still provide diagnosis accuracy.^33^^,^^34^ In some instances, ZIKV RT-PCR–positive samples were found to be cross-reactive to DENV (*N* = 16) or JEV (*N* = 8) when tested with the DEN/JEV IgM/IgG ELISA. The area under the ROC curve (AUC) value of the in-house ZIKV IgM ELISA was 0.941, reflecting excellent diagnostic accuracy. A ROC curve pointing to a cut-off line at 40 EIA units is considered optimal, providing assay sensitivity and specificity of up to 88.06% and 90.00%, respectively. The EIA unit distribution of validated samples is shown in a scatter plot with cut-off line (Figure 2). Most of ZIKV RT-PCR–positive sera samples showed higher levels of ZIKV IgM antibody binding than other groups. Some cross-reactivity among flavivirus was observed because some of DENV (mostly secondary) and JEV samples appear over the cut-off line.

### The detection of ZIKV IgM by anti-ZIKV IgM ELISAs.

The performance of the in-house ZIKV IgM ELISA was compared with two commercial ELISA kits: Euroimmun ZIKV IgM ELISA and InBios ZIKV IgM ELISA. Table 2 shows the serum panel results done by all three ZIKV IgM ELISA tests. The negative ZIKV samples were found to be 90% (72/80) negative when tested by the in-house ZIKV IgM ELISA. Fifty-nine of 67 (88.06%) ZIKV RT-PCR–positive samples were found to be positive when tested by the in-house ZIKV IgM ELISA. The Euroimmun ZIKV IgM ELISA showed 98.75% (79/80) agreement with the ZIKV RT-PCR–negative samples but only 13.43% (9/67) agreement with the ZIKV RT-PCR–positive samples, with an additional 4.48% (3/67) labeled as borderline. The InBios ZIKV IgM ELISA had a 94.03% (63/67) agreement with the ZIKV RT-PCR–positive samples with an additional one sample (1.5%) identified as “other flavivirus.” The InBios ZIKV IgM ELISA also had a 91.25% (73/80) agreement ZIKV RT-PCR–negative samples, which also includes the correct assessment of 47/60 (78.33%) as other flaviviruses.

**Table 2:** In-house ZIKV IgM, Euroimmun ZIKV IgM, and InBios ZIKV IgM ELISAs test results

ELISAs	Results	ZIKV (*N* = 67)	Others flavivirus			Negative (*N* = 20)	Total (*N* = 147)
			1° DENV (*N* = 20)	2° DENV (*N* = 20)	JEV (*N* = 20)		
In-house ZIKV IgM	ZIKV	59	2	2	4	0	147
	Negative	8	18	18	16	20	
Euroimmun ZIKV IgM	ZIKV	9	0	0	1	0	147
	Borderline	3	0	0	0	0	
	Negative	55	20	20	19	20	
InBios 2.0 ZIKV IgM	ZIKV	63	0	1	6	0	147
	Other flavivirus	1	20	18	9	0	
	Negative	3	0	1	5	20	

IgM = immunoglobulin M; ZIKV = Zika virus.

### Overall sensitivity and specificity.

Table 3 shows the case distribution and comparison of sensitivity, specificity, overall agreement, and Kappa assessment values (95% CI) of the in-house ZIKV IgM ELISA assay to two commercial ELISA kits (Euroimmun ZIKV IgM ELISA and InBios ZIKV IgM ELISA). By using ZIKV RT-PCR and DENV/JE IgM ELISA as reference standard methods, the sensitivities of the in-house ZIKV-IgM ELISA, Euroimmun ZIKV IgM ELISA, and InBios ZIKV IgM ELISA were 88.06% (59/67), 10.45% (7/67), and 94.03% (63/67), respectively. The assay specificities were 90.00% (72/80), 98.75% (79/80), and 83.75% (67/80) for in-house ZIKV-IgM ELISA, Euroimmun ZIKV IgM ELISA, and InBios ZIKV IgM ELISA, respectively. The percentage overall agreement to the ZIKV RT-PCR and DENV/JE EIA of the in-house ZIKV IgM ELISA, Euroimmun ZIKV IgM ELISA, and InBios ZIKV IgM ELISA were 89.12% (131/147), 58.5% (86/147), and 88.4% (130/147), respectively. Kappa assessment values define the in-house ZIKV-IgM ELISA (Kappa value: 0.83) and InBios ZIKV IgM ELISA (Kappa value: 0.81) as excellent, whereas the Euroimmun ZIKV IgM (Kappa value: 0.45) was classified as fair.

**Table 3:** Comparison results of In-house ZIKV IgM ELISA, Euroimmun ZIKV IgM, and InBios ZIKV IgM ELISAs

ELISA	Result*	Validation samples			Total	% Sensitivity (95% CI)	% Specificity (95% CI)	% Overall agreement (95% CI)	Kappa assessment (95% CI)
		ZIKV	Other	NEG					
In-house ZIKV-IgM	ZIKV	59	8	0	67	59/67	72/80	131/147	
	Other (a)	0	52	0	52	88.06%	90.00%	89.12%	0.83
	Negative	8	0	20	28	(77.9–94.1)	(81.3–95.1)	(83.0–93.3)	(0.74–0.91) excellence
	Total	67	60	20	147				
Euroimmun ZIKV IgM	ZIKV	9	1	0	10	9/67	79/80	88/147	
	Other (b)	3	59	0	62	13.43%	98.75%	59.86%	0.45
	Negative	55	0	20	75	(7.0–23.8)	(92.6–100)	(51.8–67.4)	(0.34–0.56) fair
	Total	67	60	20	147				
InBios 2.0 ZIKV IgM	ZIKV	63	7	0	70	63/67	73/80	130/147	
	Other (c)	1	47	0	48	94.03%	91.25%	88.44%	0.81
	Negative	3	6	20	29	(85.2–98.1)	(82.8–96.0)	(82.2–92.7)	(0.73–0.90) excellence
	Total	67	60	20	147				

IgM = immunoglobulin M; ZIKV = Zika virus.

* Other includes a) 1°, 2° dengue virus, and Japanese encephalitis virus positive of validation; b) borderline for Euroimmun; and c) other flavivirus positive for InBios ZIKV IgM ELISAs.

## DISCUSSION

Serological diagnosis of ZIKV infection is challenging, often leading to misinterpretation^35^ due to the cross-reactive nature of antibodies elicited by infection to other flaviviruses bearing common antigenic determinants or by vaccination.^20^^,^^36^ Reverse transcriptase–polymerase chain reaction is the most reliable assay today for ZIKV detection and diagnosis but is limited by the short-lived presence of viral RNA in acute serum, often lasting only 3–5 days after symptoms.^19^^,^^20^ Validated serological assays of high specificity and sensitivity, capable of detection of ZIKV-specific antibodies circulating during the acute and early convalescence phases, would expand the window of detection in support of ZIKV diagnosis and treatment.

In this study, we developed a specific in-house ZIKV IgM ELISA and measured its specificity and sensitivity using a well-defined serum panel consisting of ZIKV RT-PCR–positive serum samples and ZIKV RT-PCR–negative serum samples of other or unknown etiologies. Receiver operating characteristic curve analysis using the differences between true positive rate (sensitivity) and false positive rate (1 = specificity) showed that 40 EIA units represents the optimal cut-off point.^33^ Using this cut-off point, the AUC approximates 1 (AUC = 0.941) and indicates excellent diagnostic capabilities and accuracy of our in-house ZIKV IgM ELISA, with 88.06% sensitivity and 90.00% specificity.

We used the in-house DENV/JEV IgM/IgG ELISA^28^^,^^29^ as a model to develop the in-house ZIKV IgM ELISA. Evidence of ZIKV infection was classified when the ratio of ZIKV IgM/DENV IgM and ZIKV IgM/JEV IgM was ≥ 1.0. Using this criteria, we found ease in distinguishing between negative/primary DENV and ZIKV samples. However, we found low levels of cross-reactivity to ZIKV IgM when testing secondary DENV samples (1.49%, 1/67) or JEV (5.97%, 4/67).

The finding of negative in-house ZIKV IgM ELISA results among ZIKV RT-PCR–positive samples (8/67) could be due to improper sample collection times for IgM detection, including early collection of acute and/or late collection of the convalescent samples. Even though ZIKV cross-reactivity has been observed in the JEV samples, anti-JEV IgM read-outs were higher than anti-ZIKV IgM antibodies, facilitating assay interpretation. The JEV immunization program has been implemented in Thailand since 1990,^37^ and it has been reported that the overall protective immunity to JEV was up to 75% in Thai people.^38^ We were unable to search the medical or vaccination history of our sample donors. However, it is a reasonable expectation that JEV immunization would be a possible cause of the high anti-JEV IgM levels, which are cross-reactive and interfering with other flavivirus serological tests.^39^ Ultimately, there is a risk of false positives when ZIKV IgM ELISAs are used in areas where ZIKV co-circulates with various flaviviruses. Nonetheless, our data show that the in-house ZIKV IgM ELISA is highly specific, even when testing samples from patients with primary flavivirus infections.

Evaluations of various Zika ELISA assays have been published elsewhere.^40^^,^^41^ In general, envelop-based Zika IgM ELISA assays increase detection rates significantly over NS-1–based assays and provide a larger diagnostic window.^42^ These observations are reflected in the performance described here for the Euroimmun ZIKV IgM ELISA, a NS1-based assay with moderate results. In contrast, ZIKV IgM ELISA (using suckling mouse brain extracted crude antigen) and the InBios ZIKV IgM ELISA (recombinant envelope protein) performed substantially better. Euroimmun has recently recommended the use of the combined ZIKV IgA/IgM to improve its sensitivity.^43^

In conclusion, our study demonstrates that the in-house ZIKV IgM ELISA can be an important tool for detecting ZIKV infections in humans. The assay is an affordable and reliable option for diagnosis of ZIKV patients, especially in flavivirus-endemic countries. A limitation in the use of the in-house ZIKV-IgM ELISA is that its interpretation requires the use of DENV and JEV antibodies ELISAs, which may limit its usability in some laboratories.
